# Insulin’s Discovery: New Insights on Its Hundredth Birthday: From Insulin Action and Clearance to Sweet Networks

**DOI:** 10.3390/ijms22031030

**Published:** 2021-01-21

**Authors:** Melanie Leroux, Martial Boutchueng-Djidjou, Robert Faure

**Affiliations:** Centre de Recherche du CHU de Québec and Département de Pédiatrie, Faculté de Médecine, Université Laval, Québec, QC G1V4G2, Canada; Melanie.leroux.1@ulaval.ca (M.L.); martial.boutchueng-djidjou.1@ulaval.ca (M.B.-D.)

**Keywords:** insulin, hundredth anniversary

## Abstract

In 2021, the 100th anniversary of the isolation of insulin and the rescue of a child with type 1 diabetes from death will be marked. In this review, we highlight advances since the ingenious work of the four discoverers, Frederick Grant Banting, John James Rickard Macleod, James Bertram Collip and Charles Herbert Best. Macleoad closed his Nobel Lecture speech by raising the question of the mechanism of insulin action in the body. This challenge attracted many investigators, and the question remained unanswered until the third part of the 20th century. We summarize what has been learned, from the discovery of cell surface receptors, insulin action, and clearance, to network and precision medicine.

## 1. Introduction

**Figure 1:**
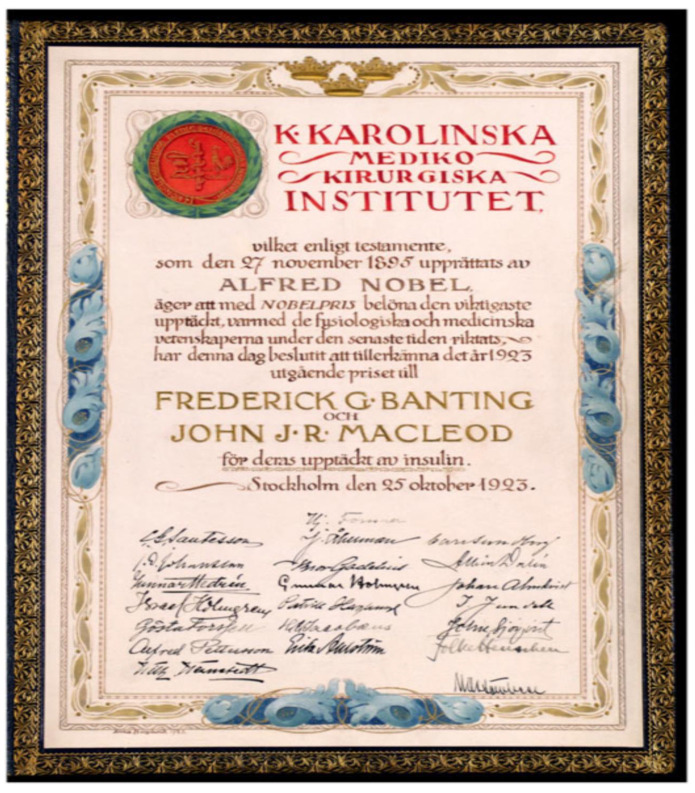
The Nobel Prize certificate to Banting and Macleod signed by the 21 members of the Nobel Prize committee. From the Nobel Foundation. In his 1925 Nobel Lecture, Macleod closed by raising the question—what is the mechanism of insulin action in the body?

The purification of insulin by the Toronto “Fab Four”, leading to the rescue of humans dying from diabetes, is one of the most dramatic examples of translational research, providing inspiration for us all. Banting, a practicing surgeon in Toronto, was inspired in October 1920 by reading an article related to pancreatic lithiasis, the islets of Langerhans and diabetes [1], and he explained his idea to Macleod in November 1920. After reviewing the experimental protocol and bypassing the pancreatic duct ligation step to preserve the islets, they started experiments with a student, Charles Best, in Macleod’s lab in May 1921. After several wrong turns, they invited Collip, a young professor from the University of Alberta on sabbatical leave in the laboratory of Macleod, who brought with him a new technique called chromatography, to join the group. Within weeks, Collip and Best developed a robust purification protocol to purify insulin from crude extracts. They successfully delivered purified insulin to the first patient, Leonard Thompson, on 23 January 1922, 15 months after the initial idea and 8 months from the onset of experiments [2,3,4,5]. They were awarded the Nobel Prize in October 1923 (Figure 1). In medical research, the ultimate goal is to have a practical application introduced rapidly after the relevant basic science discovery. Albeit unachievable now—that was translation!

One hundred years later, we have learned that insulin is not fully restorative [6]; many patients on insulin suffer complications, and strict attention to a dietary program is essential. Normalized insulin remains the mainstay for treating type 1 diabetes (T1D) [7]. It was discovered that diabetes is a leading cause of morbidity and mortality, with type 2 diabetes (T2D) accounting for more than 90% of cases [8]. Insulin is also prescribed for patients with T2D who are not achieving adequate control of hyperglycemia with drugs, such as Metformin and other interventions [9]. Insulin is used for the control of gestational diabetes [10]. It is used to reduce transient hyperglycemia, which in nondiabetic patients can accompany acute medical situations [7]. The idea of a strong hereditary component in both T1D and T2D became well established. Approximately 40% of the variance in T2D is due to genetics [11,12], with an association between parental history deriving from inherited traits but also environmental factors [13]. The genetic analysis of large cohorts, mainly originating from genome wide association (GWAS) data, complemented by analyses of exome- and genome sequence data, has raised questions concerning the “missing heritability” and the biological space occupied by numerous common variants, with a small causality dispersed throughout the human genome and many of them located in regulatory intronic areas [14,15,16,17].

**Figure 2:**
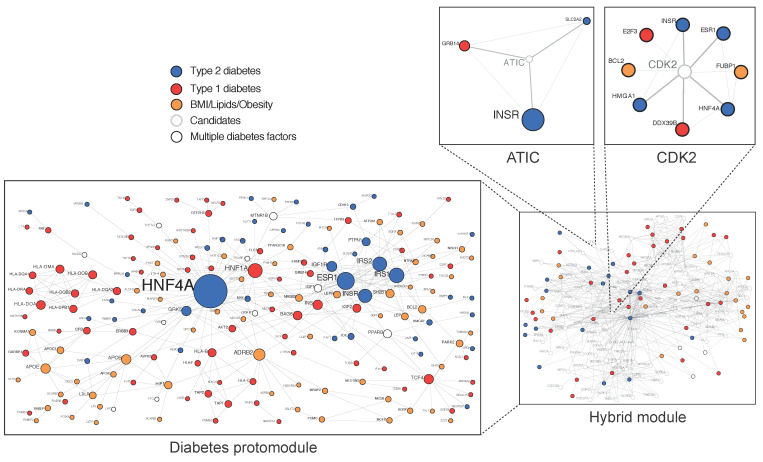
Diabetes-associated genes form a protomodule. 184 genes at risk of T2D are here validated in a single component proteins–proteins interaction network of 309 iterations called protomodule. The general topology of the protomodule is characteristic of a disease network with the presence of few central hubs of a large size, surrounded by numerous peripheral hubs of a smaller size. Expansion of protomodule (seeds) through physical interactions with gene products in IR-containing endosomal network (IREN) results to a hybrid module where each protein from IREN, connected to at least three “seeds” from the protomodule, is considered as a gene candidate (grey nodes) for T2D. Candidates were additionally validated using gene ontology analysis for subcellular colocalization with connected “seeds” from the T2D protomodule and experimental approaches. (Adapted from Boutchueng et al. *PLoS ONE* **2018**, *13*, e0205180).

Because all cellular processes involve scale-free networks, their study at a network level has been an important recent focus in cell biology and medicine and can be used to understand the genotype/phenotype relationship [18,19,20]. In 2007, the first diseasome constructed by the mathematician Albert Barabasi from the Online Mendelian Inheritance in Man database (OMIM) showed that T2D occupies a central hub [21]. In 2018, the first T2D physical protein–protein interactions network (PPIN), constructed from validated variants, termed diabetes protomodule, displayed the proteins’ hepatic nuclear factors HNF4A and HNF1A as central nodes [22] (Figure 2). The HNF1A variant identified by whole-exome sequencing in a homogeneous Latino cohort is an example of a rare variant with a high causality at a population level [23].

## 2. The Insulin Receptor-Tyrosine Kinase (IR)

Macleod closed his Nobel Lecture speech by referring to the “perplexing problem of the mechanism of insulin action in the body” [24]. The answer was provided 50 years later. Instead of asking “what is the mechanism of insulin action?” the advent of radio-immunoassays allowed researchers to ask the question, “how does the cell know that insulin is there?” The concept of cell surface receptors having the capacity to detect a faint signal in a high background was introduced [25]. Insulin without its receptor cannot perform any functions [26]. Soon after the discovery of tyrosine phosphorylation [27], it was demonstrated that the mature IR is a tyrosine kinase [28,29,30]. The Kadowaki variants, which are located in the IR tyrosine kinase domain and have resulted in severe insulin resistance in a child with Leprechaunism syndrome [31], are examples of rare variants with a high causality. The structure of the mature, full-length IR in complex with insulin was recently resolved by cryo-electron microscopy and allowed for a revision of the IR activation model [32]. Notably, following the binding of two insulin molecules to the inversed V-shaped extracellular alpha-subunits, two hinge motions were confirmed. The first one involves a large conformational change between the cysteine rich (CR) and leucin rich-2 (L2) domains, where the loop that connects the two domains acts as the hinge. The second one occurs around the linker between the L2 and fibronectin (FnIII-1) domains, resulting in a new interdomain interaction between specific residues of L2 and FnIII-1. The combination of these two hinge motions makes each of the IR promoters adopt an inverted “J” shape. Two J-shaped promoters constitute the “T”-shape active tetrameric IR. Following the formation of the T-shaped IR, the membrane-proximal regions of the two IR promoters are brought together by an interaction between the two FnIII-2 domains. These large mechanical movements, similar to the epidermal growth factor (EGF)receptor model, promote the transmembrane TM–TM interaction and enable the trans-autophosphorylation of the intracellular tyrosine kinase domains, leading to the active IR [32]. Of further interest, the IR seems to have two different conformations at low and saturating insulin concentrations, promoting different activities and suggesting that the IR may act as a sensor of circulating insulin concentration. The different conformational states induced by low or high levels of circulating insulin would generate various signaling outputs (metabolic versus mitogenic responses), implying that insulin levels should be finely regulated. With a single conformational change (two insulin versus four molecules bound by a receptor), the IR can generate a regulated response, and the local membrane microenvironment seems to also be important [33,34]. The IR has two isoforms (IR-B and IR-A) [35]. The vast majority of studies involved in the mechanisms of regulation of endocytosis and sorting of the receptor were performed before the identification of the IR-A isoform, and mostly in adipocytes, which preferentially express the IR-B isoform [36].

## 3. IR Endocytosis and Insulin Clearance

Following insulin binding on the cell surface of hepatocytes, the IR insulin complexes are internalized and insulin is degraded [37]. Najjar and colleagues identified the molecular mechanisms by which CEACAM1 regulates insulin internalization and degradation through the IR-A isoform in the liver [38,39,40]. On the other hand, Mirsky and Broh-Kahn first described the existence of an insulin-degrading enzyme (IDE) that inactivated insulin in rat tissue extracts from the liver [41]. Roth and colleagues purified to homogeneity this protease, characterizing its biochemical properties and identifying that insulin can bind to IDE [42,43]. Duckworth and colleagues identified a peptide bonds cleavage by IDE in vitro and in cultured cells and postulated a model in which IDE played a major role for cellular handling and the degradation of insulin [44,45].

**Figure 3:**
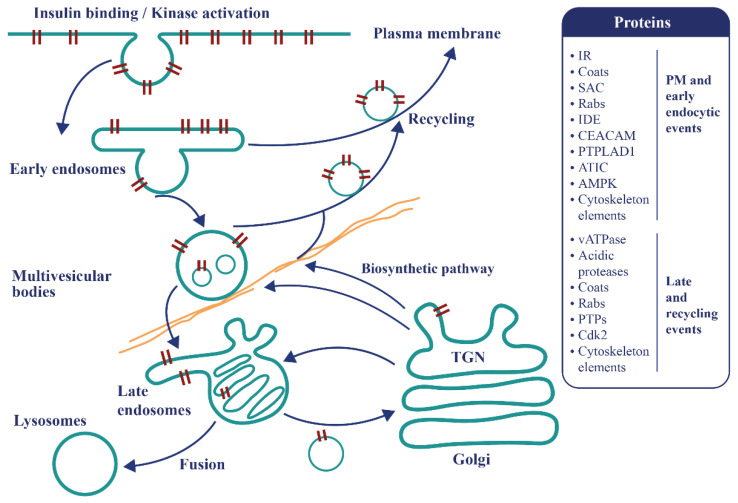
The endosomal apparatus is a topologically versatile tubulo-vesicular compartment controlling signaling events and metabolism. Following insulin binding, the tyrosine kinase-activated insulin-IR complexes are immediately internalized as endosomes. At this locus, a decision is made to recycle the insulin free-IR back to the cell surface or to transport the active tyrosine kinase-activated complexes towards late compartments for late signaling, recycling or degradation. Some proteins present in the T2D disease module and involved in these mechanisms are represented.

Endosomal apparatus is a topologically versatile tubulo-vesicular compartment controlling signaling events and metabolism [46,47] (Figure 3). Recent findings promote a re-examination of the mechanisms of IR endocytosis and its relationship with insulin resistance, as well as clearance and its connection with pancreatic secretion. A specific internalization pathway involving subunits of the mitotic check point complex (MCC) was described [48]. After insulin stimulations, the mitosis arrest deficiency 2 (MAD2) and budding uninhibited by benomyl 1-related 1 (BUBR1), as well as the clathrin adaptor protein AP2, assemble at the IR-beta subunit, triggering the internalization of the active IR complexes and stopping insulin signaling at the plasma membrane [48]. The MAD2-binding protein p31comet (P31^comet^) prevents the conformational activation of MAD2 and plays a critical role in insulin signaling. MAD2 directly binds to the C-terminal MAD2 interacting motif (MIM) of the IR and recruits AP2 to IR through BUBR1. P31^comet^ prevents IR endocytosis by blocking the interaction of BUBR1-AP2 with IR-bound MAD2. Adult liver-specific P31^comet^ knock out mice exhibit premature IR endocytosis in the liver and whole-body insulin resistance [49]. Conversely, BUBR1 deficiency delays IR endocytosis and enhances insulin sensitivity in mice, and a reduction of IR on the cell surface in liver samples of T2D patients was reported [50]. In addition to MAD2, BUBR1, and P31^comet^, it was shown that a cell division cycle of 20 proteins (cdc20) is required for IR endocytosis. Feedback regulation of IR endocytosis through a regulation switch on an insulin receptor substrate 1/2 (IRS½) that is dependent on a SH2 domain-containing protein tyrosine phosphatase (PTP), SHP2, and the MAPK pathway was identified. In addition to the MIM motif, the IR juxta-membrane Y960 motif, required to bind IRS1/2, in turn recruits AP2 through YXX Φ motifs collaborating with the MAD2-cdc20-BUBR1 module and leading to efficient formation of the clathrin cage [50]. It was also confirmed that MAD2 disappears from the rat liver plasma membrane within seconds following injections of insulin at fixed time points, further supporting the idea that MCC components, which control the fidelity of chromosome segregation during mitosis, are repurposed in the interphase for early events of IR endocytosis [22]. In addition, there is no concomitant appearance of MAD2 in endosomes, indicating that MAD2 complexes rapidly dissociate as expected for clathrin coats [22]. This supports the view that dysregulation in endocytosis is related to insulin resistance. Of further interest, BUBR1 insufficiency causes premature aging [51,52], raising the question of whether IR might reciprocally act on MCC and spindle assembly checkpoint (SAC) signaling during mitosis.

As for the EGFR, IR tyrosine kinase activity appears to be the crucial regulator selecting ligand-dependent movements and signaling in endosomes [53,54,55]. Sorting is achieved with tubulovesicular compartments whose contents are modified by the entry and exits of 70–80 nm vesicles according to the conversion model [56,57]. Proteins involved in these processes have multiple YR-tyrosine phosphorylation regulatory motifs, including Rab5c and vATPase subunits [22]. For example, the lumenal dissociation/degradation sequence of insulin occurring at an acidic pH generated by the proton pumping activity of the vacuolar ATPase (vATPase) would be affected [22]. The liver parenchyma is a major site for insulin clearance, and defects in this process can cause hyperinsulinemia and secondary resistance [58,59,60]. Liver-specific knockout of IR in mice and mice deleted of CEACAM1 develop hyperinsulinemia [60], implying that insulin action and production are essential endosomal responses that limit anabolic processes to nutrient oversupply.

## 4. From PPIN to a T2D Disease Module

Proteins assemble with each other to form stable complexes or transient and regulated interactions, for instance in signaling mechanisms [61]. The proteasome, which includes several subunits that maintain proteostasis, is an example of stable complexes having a specialized function [62]. Examples of transient association include signaling mechanisms initiated by cell surface receptors such as the IR, which signals the presence of insulin through a series of phosphorylation events that culminate in the binding and activation of lipid modifiers, phosphorylation, activation, compartmentation of kinases such Akt, and downstream responses [54].

One major question of interest regarding genetic perturbations is how variations observed in human disease genes may affect PPIN architecture and how PPINs are rewired during evolution and under modern nutritional conditions. It was shown in the eukaryotic *Saccharomyces Cerevisiae* model that PPINs are conserved and particularly sensitive to gene dosage effects, by haploinsufficiency for example, or by defects in physical protein-protein interactions. Mechanisms of PPIN modulation were classified into two main groups. The first type of mechanism acts through changes at the levels of transcription, translation, post-translation modification and cellular compartmentation. These layers of regulation affect the stoichiometry of binding partners and, hence, the amount of complex formed. The other mechanisms act directly on the proteins independently of their abundance and can directly affect the binding between two proteins. By far, the best-characterized mechanism among these is transcriptional regulation [63]. This is important for T2D as a good part of the numerous common variants is located in intronic regulatory sequences [17]. It was also demonstrated that yeast phosphoproteins have more interacting partners than non-phosphoproteins [64,65,66], with over 46% of the 628 PPINs found reported to be phospho-tyrosine-dependent [67]. The high-throughput tools available for yeast have also allowed testing for the effect of genetic perturbations on PPINs on a large scale. It was found that different mutant alleles of the same protein caused different perturbations on their PPIN profiles. Some alleles mimicked the effects of a complete protein removal while others caused PPIN-specific changes, referred to as edgetic perturbations [68]. Indeed, it was revealed that mutations affecting the same protein, but causing distinct diseases, could be explained by the distinct structural properties of the proteins resulting from mutant alleles. Mutations leading to loss of interactions tended to affect buried residues, while mutations affecting only a subset of PPINs tended to lie on the surface [63]. Altogether, these studies provide insights into reconciling the phenotypic consequences of disease alleles with their genotype and phenotype. In another study, where approximately 2500 mutant alleles of proteins implicated in human diseases were tested, 40% of the detected PPINs were perturbed by the tested mutations. It was concluded that allele variants not implicated in diseases generally retained all their PPINs, contrary to disease-associated alleles and the fact that gains of interactions rarely happen [69]. Altogether, the results suggest that the cause of diseases associated with one-third of the mutations may involve perturbations of PPINs or other mechanisms, such as localization changes, rather than a complete loss of protein function. These findings illustrate the power of associating genotypes with phenotypes at a PPIN level. In humans, variants of unknown significance (VUS) can be associated with their effects on PPINs [70,71], and the predictive power of the notion of the disease module at a whole interactome level was first illustrated in asthma [72].

A PPIN view of IR-containing endosomes revealed the presence of a module where variants associated with T2D genetic risk converges and the cell cycle regulator Cdk2 represents the central hub [22] (Figure 3). The hypothesis-free T2D disease module forces reflection on several questions concerning noncanonical insulin-dependent mechanisms, aiming to comprehend the primary mechanisms by which cells make decisions when facing changes in environmental conditions and how genotyping changes map onto phenotypes. Important questions concerning the T2D disease module are how rare and common variants change their organization and contribute to disease and how they are related with other diseases. Affiliated questions are how architecture changes during evolution and how endosomes drive a conserved homeostatic response, which can be functional when avoiding excessive nutrient accumulation inside the cells in modern over-nutrition conditions. Co-functionality with islets, insulin resistance and clearance is another affiliated question, with applications for T2D cohort subgrouping and precision medicine [73].

Several observations indicate that the T2D module hidden in endosomes is particularly robust: (i) random PPIN reiterations show that they cannot arise by chance (*p*-value < 0.0049) [22]; (ii) a larger integrated view of T2D GWAS signals and PPIN constructed at a lower probability (4 × 10^−^^5^) independently identified Cdk2 as a major causal gene [74]; (iii) mice deleted for Cdk2 are viable [75,76], and mice with pancreas-specific deletion of Cdk2 are glucose intolerant, primarily due to defects in insulin secretion [77]; and (iv) Cdk2 is responsive to insulin in the liver plasma-membrane, and Golgi/endosomes fraction and regulate vesicular fusion events [78]. With the view that during cell division endocytic events cease [79], as seen for cell division checkpoints, this supports the idea of local action performed by MCC components streaming from the cell surface to early and late hepatic IR trafficking events in a “pause to decide” strategy. The role of Cdk2 in insulin secretion also supports the co-functionality of the T2D disease module with pancreatic islets.

Most common diseases have a polygenic architecture fully consistent with conserved biological systems supported by multiple back-up mechanisms. Specifically, essential genes are described as having more PPIN partners and are more centrally located in a network [80]. Several independent methods are available to define essentiality, including exploration of the relationship between genotype and phenotype in the mouse model [81], conservation among species [82,83], and human cell stiffness [84]. Human gene essentiality was first associated with the study of Mendelian diseases, which generally reflect the consequence of severe genetic lesions on human fitness [21,85]. In marked contrast with Cdk2, which is not considered essential on the basis of non-lethality in the mouse model [75], the metabolic enzyme ATIC, an important node in the T2D disease module [22,86], is an example of a highly conserved enzyme present in prokaryotes but not considered essential in terms of lethality in the mouse model [81]. ATIC variants are, however, responsible for a severe AICA-ribosiduria syndrome in humans [87]. In a recent long-term survey, multiple episodes of idiopathic transitory hypoglycemia were reported for one case of AICA-ribosiduria. These events might be related to increased activation of the insulin pathway in hepatocytes in the context of ATIC deficiency. The involved variant (Ala136Thr) is located in the cyclohydrolase domain next to Lys 137, an amino acid crucial for the cyclohydrolase activity of dimeric ATIC [88]. The well-positioned Ala136Thr variant could thus drive metabolic comorbidities by decreasing ATIC connectivity in the PPIN.

V-ATPase subunits are conserved in eukaryotes and play an important role in hepatic insulin clearance by promoting the insulin dissociation/degradation sequence of internalized IR complexes [53]. Proton pumping activity also promotes destabilization of the CEACAM1/fatty acid synthase (FASN) complexes, which are key regulators of insulin clearance [60]. Hepatic insulin clearance coordinates, in concert with insulin secretion, the regulation of insulin homeostasis [54], further highlighting the co-functionality of the T2D disease module with islets. The 3-OH acyl-CoA dehydratase3 (hacd3) gene is not essential but is considered a Golgi fitness gene in humans [73]. The hacd3 gene product, also termed PTPLAD1, is highly responsive to insulin in vivo and is part of the endocytosed IR complexes, where it undergoes tyrosine phosphatase activity [22,86]. Hacd1–4 proteins are involved in the third step of very long chain fatty acid (VLFA) synthesis [89]. VLFAs are involved in microdomain formations, organelle structure, and signaling [90,91]. Hacd3 is more highly expressed in the brain, liver, kidneys and placenta. Its VLFA elongation activity is, however, not or barely detectable in overexpression conditions, and its role is unclear [92]. Its insulin responsivity suggests a recent, specialized role in the regulation of IR complexes and insulin clearance through its tyrosine dephosphorylation activity, possibly complementing the activity of CEACAM1/FASN complexes. Of equal interest, the insulin index and pancreatic insulin granules were recently found to have decreased in diabetic rats with increased V-ATPase expression in their islet cells, inhibition of V-ATPase inhibited renal gluconeogenesis enzymes, and improved insulin secretion [93], further linking the network view and co-functionality with islets.

The need for system biology approaches was formulated by cell biologists–“if we do not understand the normal cell, we cannot understand the disease”–implying the aid of experts in various areas of the sciences, including cell biology, physics, mathematics, biomedical sciences, and clinical research, is necessary [94]. In this first quarter of the 21st century, bioinformatics does not yet allow a network view of the whole cell and organism. The endosomal apparatus represents less than 1% of the total cell and can be represented in a real network that unmasks new cellular mechanisms where numerous variants converge. A key assumption of the T2D disease module concerns the hypothesis-free identification of layers of noncanonical, essential homeostatic pathways. While appealing, this may underestimate the pressure of the modern overnutrition conditions that often result to insulin resistance in adipocytes. This results in the release of inflammatory fatty acids and other late, non-conserved signaling events unrelated to insulin-dependent mechanisms [95]. According to the examples documented above, the T2D disease module is composed of a core of highly conserved genes and stiffness genes. Reconsidering the essentiality and fitness [96,97,98,99,100] of the T2D module and its associated primary mechanisms will be important to achieve the goals of network and precision medicine [101]. The presence of hubs involved in histones and DNA post-translational modifications also implies that the T2D disease module, by effecting histones and DNA codes, is informative in terms of predisposition to adverse epigenetic events.

Diabetes is presently classified into two main categories, type 1 and type 2 diabetes, but type 2 diabetes is particularly heterogeneous in terms of genetics, clinical presentation and outcomes. An important goal for clinicians and researchers is to classify subtypes of diabetes in order to predict clinical complications and more accurately select therapies. Clustering can be achieved with the integration of a T2D disease module that helps to link to a primary mechanism for each group of patients (insulin signaling response, clearance and production in islets according to the beta cells centric model). It would be then possible to subclassify small cohorts of patients to help diabetologists in their day-to-day practice [73].

In terms of the Macleod question–what is the mechanism of insulin action in the body?–the actual answer is provided by the presence of a physical and functional PPIN, where hereditary traits converge also integrates epigenetic predispositions and is sensitive to modern overnutrition conditions.
